# Quality of life of dermatology outpatients and its associated factors in Halibet National Referral Hospital in Asmara, Eritrea

**DOI:** 10.1038/s41598-024-67224-1

**Published:** 2024-07-15

**Authors:** Tomas Tewelde, Nuru Abdu, Dawit G. Weldemariam, Natnael Bereket, Mulugeta Russom, Eyasu H. Tesfamariam

**Affiliations:** 1 Pharmacy Unit, Department of Medical Sciences, Orotta College of Medicine and Health Sciences, Asmara, Eritrea; 2 Department of Pharmacy, Adi-Tekelezan Community Hospital, Ministry of Health, Adi-Tekelezan, Eritrea; 3 Department of Pharmacy, Hazhaz Zonal Referral Hospital, Ministry of Health, Asmara, Eritrea; 4 Eritrean Pharmacovigilance Centre, National Medicines and Food Administration, Ministry of Health, Asmara, Eritrea; 5 Department of Medical Informatics, Erasmus Medical Centre, Rotterdam, The Netherlands; 6 European Program for Pharmacovigilance and Pharmacoepidemiology, University of Bordeaux, Bordeaux, France; 7 Biostatistics and Epidemiology, Department of Statistics, College of Sciences, Mai-Nefhi, Eritrea

**Keywords:** Quality of life, Skin disease, Dermatology, Associated factors, Skindex-29, Eritrea, Psychology, Diseases, Health care, Medical research

## Abstract

Skin diseases are prevalent globally and can have detrimental effects on the individual’s health-related quality of life (HRQoL). The treatment of dermatological patients typically focuses on clinical signs and symptoms and a subjective view of the impact of the disease on the patient’s life. Assessing quality of life can help provide patients with better service, by acknowledging their real needs and interfering with treatment decisions. The aim of the study was therefore to assess quality of life of dermatology outpatients and its associated factors. An analytical cross-sectional study was conducted in the dermatology clinic of Halibet National Referral Hospital in Asmara, Eritrea. HRQoL data were collected between May 6 and August 18, 2022 using a validated standard tool (Skindex-29). Descriptive statistics, logistic regression and paired t-test were employed using Statistical Package for Social Sciences (Version-26.0). A total of 375 dermatology clinic out-patients with a median age of 29 (Interquartile range: 25) were included in the study. The most commonly seen skin diseases were eczema, seborrhoeic dermatitis and tinea pedis. Emotion, symptom, and functioning domains of HRQoL were severely impaired in 75.7%, 50.4% and 57.6% of all dermatology outpatients, respectively. More than half of the respondents (57.9%) had a severely impaired overall HRQoL. In the overall HRQoL, being a rural resident [Adjusted Odds Ratio (AOR) 1.98, 95% CI 1.18, 3.33] and presence of chronic illness (AOR 2.16, 95% CI 1.22, 3.82) were significantly associated with severely impaired overall quality of life. A significantly higher mean score (*p* < 0.001) was observed in emotion [Mean (M) = 55.60, Standard Deviation (SD) = 21.0] as compared to functioning (M = 46.89, SD = 21.2). On the other hand, significantly higher mean score (*p* < 0.001) was observed on symptom (M = 54.08, SD = 20.5) as compared to functioning (M = 46.89, SD = 21.2). Skin diseases severely affected the emotion, functioning, and symptom domains of health-related quality of life. This highlights the importance of providing physical and psychosocial support to patients with dermatologic problems.

## Introduction

Skin diseases are commonly reported health conditions with a significant global public health burden and considerable disability^1^. Patients with skin diseases may experience severe symptoms such as itching, pain, and discomfort, which can have a profound psychological impact. Moreover, patients with severe dermatologic conditions and lower disease-specific health-related quality of life are more likely to seek extensive medical services^2^. Doctors are often focused on accurate diagnosis and appropriate treatment of patients’ skin conditions. Although they are aware of the devastating effect of patient’s skin condition on their lives, their quality of life, and the economic and social impact of their disease may earn little attention which could further complicate the condition^3^. Hence, doctors are encouraged to closely pay attention to the improvement of overall well-being of their patients, as different studies has revealed that, the severity of skin conditions are found to be associated with severely impaired quality of life^3–7^. Many skin conditions, such as psoriasis, eczema, vitiligo, acne vulgaris, and cutaneous lupus erthyromytosis are rarely life threatening, but they can negatively affect patient’s quality of life^8–10^.

According to a study of the global burden of disease project, skin diseases are responsible for causing 41.6 million disability-adjusted life years making them the fourth leading cause of non-fatal morbidity worldwide^11,12^. Chronic skin diseases have long-term negative effect on patients’ health-related quality of life, thus it is paramount to have a better understanding of the effect of these diseases and their determinants^8^. Several risk factors are associated with impaired health-related quality of life^13,14^. The interaction of skin conditions and their psychological effect, however, is complex, and difficult to identify the root causes. Patients’ self-image, identity, socialization, sexuality, perception and fear of their disease conditions among others were found to be determinants of the overall quality of life of patients attending dermatology clinic^15–17^. It is also important to note that the diagnosis of skin condition and its chronicity were also identified as determinants of the patients’ quality of life^15^. Despite the above-mentioned facts, management of skin diseases usually focuses on clinical signs and symptoms and on a subjective view of the impact of the disease on the patients’ life^18^. According to the World Health Organization, quality of life (QoL) is defined as the perception of individuals of their current situation considering their culture and their relationship value system with their goals, expectations, standards and concerns, and their impact on physical health, mental conditions and independency of social relationships^19^. QoL measures can potentially allow dermatologists to record and monitor a patients progress with valid and reliable methods that allow intrapersonal and interpersonal comparisons^3^.

Assessing QoL is important to identify individual, societal and institutional drivers for skin condition-related health problems and guide policy decisions on the risk mitigation plans that need to be in place. No previous study was conducted in Eritrea to measure the quality of life of patients with a wide range of skin conditions. The aim of the study was therefore to assess quality of life of dermatology outpatients and its associated factors in the dermatology clinic of Halibet National Referral Hospital.

## Materials and methods

### Study design and setting

An analytical cross-sectional study with a quantitative approach was conducted in the dermatology clinic of Halibet National Referral Hospital in Asmara, Eritrea. It is the only national referral hospital with a well-established dermatology department providing services to patients with various skin diseases. In Eritrea, healthcare services are highly accessible and affordable, provided at heavily subsidized or nominal costs through the public health system. The healthcare delivery model is structured on a three-tier basis: the primary level consists of health stations, health centers, and community hospitals, serving as the initial point of contact for patients. The secondary level comprises regional referral hospitals and second-contact hospitals within sub-regions, to which patients are referred from the primary level. The tertiary level is made up of national referral hospitals located in the capital city, Asmara. Patients can be referred to higher-level facilities by their healthcare providers, or they can be self-referred directly to secondary or tertiary hospitals if they deem it necessary. This allows for a streamlined and accessible healthcare system that caters to the diverse medical needs of the population. Data were collected from May 6 to August 18, 2022 for a period of 90 working days.

### Source and study population

All outpatients attending the dermatology clinic during the study period were the source population. Patients aged 18 years and above who were diagnosed with any skin diseases and willing to participate formed the study population.

### Sample size and sampling technique

The sample size was computed by considering the finite population correction factor formula: n = NZ^2^pq/ [pqZ^2^ + (N−1)d^2^]. The total sample size (n) was calculated using the following assumptions: expected proportion of patients whose QoL was severely affected by skin disease (p) and those whose QoL is not severely affected by skin disease (q) were taken as 0.5, Z statistic for 95% level of confidence (Z = 1.96), estimated population size (N) of 2700, margin of error (d) of 0.05 and 10% non-response rate. Considering the above assumptions, the final sample size was 375. A systematic random sampling was employed to select subjects from the study population.

### Data collection tool and approach

A structured and assisted self-administered data collection tool was used to collect data. The data collection tool had two sections. Section A was intended to record the socio-demographic and background characteristics of the patients such as age, sex, educational level, religion, ethnicity, occupation, and presence of chronic illness. Section B contained a standard and validated tool (Skindex-29) to assess the patients’ health-related quality of life (HRQoL). Skindex-29 consists of 30 items, of which 29 items (with the exception of item 18: side-effects of treatments) are assigned in three domains such as emotions (10 items), symptoms (7 items) and functioning (12 items). Permission to use Skindex-29 was granted from MAPI Research Trust (the organization holding the copyright for the use of the tool). Evidence suggested that this tool exhibits good psychometric properties in terms of measuring HRQoL^20^. The data collection tool was translated into local language (Tigrigna), translated back to English to assure consistency and finally to Tigrigna by three language and literature experts.

The investigators explained purposes of the study to the participants during their dermatology outpatient visit and those who gave written consent were enrolled in the study. Diagnosis of the skin disease was confirmed by two experienced physicians working in the outpatient dermatology clinic. After confirming the diagnosis, the data collection tool was self-administrated to each patient and filled in a separate room within the dermatology clinic. Besides, a face-to-face interview was conducted for those patients with no formal education and one patient was interviewed at a time to ensure privacy. During submission, investigators checked the filled questionnaire for completeness.

### Variables

Quality of life of the dermatology outpatients was considered the dependent variable and the independent variables were socio-demographic and background characteristics such as age, sex, educational level, religion, ethnicity, occupation, and presence of chronic illness.

### Variable measurement and interpretation

Quality of life was measured with Skindex-29 using 5-point Likert scale ranging from ‘never’ to ‘all the time’. As per the scale guideline, a score of ‘0’, ‘25’, ‘50’, ‘75’ and ‘100’ was given to the responses ‘never’, ‘rarely’, ‘sometimes’, ‘often’ and ‘all the time’, respectively. A scale score was computed by dividing the mean of a patient’s responses by the items in a given scale. On the other hand, as per the scale guideline, missing of more than 25% of responses to an item, missing of more than 25% of the items in a domain, and an item with multiple answers (considered as missing item) were not present in this study. Higher score within domains illustrated a higher impact of the skin disease on the patients’ quality of life.

According to a literature, the Skindex-29 cut-off scores for severely impaired HRQoL were recognized and showed to have the highest accuracy^3^. As such, the cut-off scores for severely impaired HRQoL of ≥ 52 points on symptoms, ≥ 39 on emotions, ≥ 37 on functioning, and ≥ 44 on the total score were used in this analysis.

### Quality assurance

The data collection tool (along with Tigrigna Version of Skindex-29) was peer-reviewed by a panel of experts in the fields of medicine, pharmacy, pharmaco-epidemiology, public health, and epidemiology to verify face and content validity. The data collection tool was then modified as per the comments given by the experts and subject to a pre-test. The pre-test was conducted on 38 participants from 13 to 16 April, 2022 in the dermatology outpatient clinic of Halibet National Referral Hospital. It aided in assessing translation accuracy, estimation of time needed to complete the questionnaire, and familiarizing data collectors. A two-day training workshop was provided for data collectors to clearly understand the study objectives, questionnaires, and approach of conducting the face-to-face interview. To ensure reliability, two pharmacists, NB and TT, who had prior similar experience were selected and oriented to conduct the data collection so as to understand the within and between inter-rater uniformity.

### Statistical analysis

The collected data were entered using CSPro (version 7.0), cleaned and exported to IBM Statistical Package for Social Sciences (SPSS) [Version 26.0] for statistical analysis. Descriptive analysis was performed using percentages and mean (Standard Deviation (SD)). The scores of the domains of QoL were computed from the respective items and converted to 100%. Furthermore, the scores were categorized into binary as per the guidelines of the Skindex-29 tool. The factors that affect the domains of QoL and total QoL binary responses were assessed primarily using bivariate logistic regression. Factors that were significant at bivariate were retained for multivariable logistic regression and adjusted odds ratios (AORs) were computed consequently as a measure of association. To determine which domain of the QoL is highly affected, paired t-test was employed for comparisons. Tables and figures were used to present the results. The level of significance for all variables was tested at *p* value less than 0.05.

### Ethical approval and consent to participate

Ethical approval was obtained from Ministry of Health (MOH) research ethics and protocol review committee (reference number: 03/09/2019). Besides, permission was obtained beforehand from the medical director and head of outpatient department of the dermatology clinics of Halibet national referral hospital where the actual study and the pre-test were conducted. Study participants were informed about the objectives of the study and written informed consent was obtained before enrollment into the study. Information obtained from this study was kept confidential and used only for this study’s purpose. This study conforms to the principles outlined in the Declaration of Helsinki (2013).

## Results

### Socio-demographic and background characteristics

A total of 375 participants with a median age of 29 (IQR: 25) were enrolled in the study. Two-hundred and one participants (53.6%) were males. The majority of the participants were urban inhabitants (77.3%), aged between 18 and 40 years (67.2%), and completed their secondary education (49.6%). Moreover, 56% of the participants were employed, and 81.6% of them had no chronic illness (Table 1). Detailed socio-demographic and background characteristics of the study population are depicted in Table 1.

**Table 1:** Socio-demographics and background characteristics of study participants (N = 375).

Variables	Category	Frequency	Percentage
Age (Md; IQR = 29, 25)	18–40	252	67.2
	41–59	82	21.9
	60 and above	41	10.9
Sex	Male	201	53.6
	Female	174	46.4
Educational level	No formal Education	40	10.7
	Primary	45	12.0
	Junior	22	5.9
	Secondary	186	49.6
	Higher	82	21.9
Residence	Urban	290	77.3
	Rural	85	22.7
Ethnicity	Tigrigna	340	90.7
	Others*	35	9.3
Occupation	Employed	210	56.0
	Unemployed	105	28.0
	House wife	60	16.0
Chronic illness^‡^	Hypertension	13	3.5
	Asthma	11	2.9
	Diabetes	3	0.8
	Others^‡^	42	11.3
	No chronic illness	305	81.6

*Md* median; *IQR* interquartile range.

*Others include Tigre, Bilen, Saho and Afar.

^‡^Others include Human Immunodeficiency Virus, Cancer and Goiter.

^‡^Total percent might exceed 100 due to multiple answers.

Of the 375 participants, a total of 516 skin diseases were documented. The most commonly observed skin diseases were eczema (10.1%, n = 52), seborrhoeic dermatitis (7.9%, n = 41), tinea pedis (7.2%, n = 37) and acne vulgaris (5.6%, n = 29) (Table 2).

**Table 2:** Distribution of common skin diseases seen among the study participants (n = 516).

Skin diseases	Frequency	Percentage
Eczema	52	10.1
Seborrhoeic dermatitis	41	7.9
Tinea Pedis	37	7.2
Acne vulgaris	29	5.6
Palmoplantar hyperkeratosis	22	4.3
Psoriasis	22	4.3
Plantar hyperhidrosis	20	3.9
Onychomycosis	18	3.5
Lichen planus	18	3.5
Pityriasis versicolor	18	3.5
Vitiligo	15	2.9
Melasma	12	2.3
Tinea Corporis	12	2.3

### Quality of life of the dermatologic outpatients using Skindex-29

**Figure 1:**
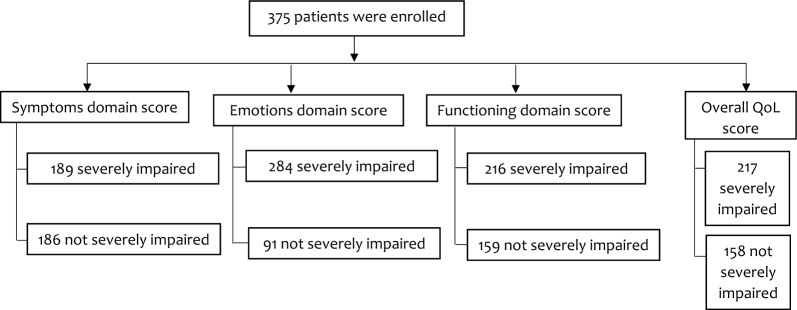
Quality of life domains score (Skindex-29). *QoL* quality of life.

In the emotion domain, majority of the respondents (n = 260/375, 69.3%) reported that they frequently worried that their skin condition might get worse. Moreover, most of them (61.6%) frequently worried about getting scars from their skin conditions. However, only one in five of the participants (19.5%) reported that they frequently get frustrated by their skin condition (Table 3). The mean (SD) score of the emotion domain was 55.60 (21.0) out of 100. A total of 284 participants had a severely impaired emotion domain (Fig. 1).

**Table 3:** Percentage distribution of quality of life domains within Skindex-29 (N = 375).

Items of quality of life domains	How often the skin disease affects a patient		
	Never/rarely n (%)	Sometimes n (%)	Often/all the time n (%)
Emotion			
I worry that my skin condition may be serious	124 (33.1)	28 (7.5)	223 (59.5)
My skin condition makes me feel depressed	186 (49.6)	48 (12.8)	141 (37.6)
I worry about getting scars from my skin conditions	118 (31.5)	26 (6.9)	231 (61.6)
I am ashamed of my skin condition	219 (58.4)	27 (7.2)	129 (34.4)
I worry that my skin condition may get worse	83 (22.1)	32 (8.5)	260 (69.3)
I am angry about my skin condition	221 (59)	37 (9.9)	117 (31.2)
I am embarrassed by my skin condition	240 (64)	23 (6.1)	112 (29.9)
I am frustrated by my skin condition	272 (72.5)	30 (8.0)	73 (19.5)
I am humiliated by my skin condition	249 (66.4)	26 (6.9)	100 (26.7)
I am annoyed by my skin condition	224 (59.7)	40 (10.7)	111 (29.6)
Symptoms			
My skin hurts	180 (48)	61 (16.3)	134 (35.7)
My skin condition burns or stings	158 (42.2)	50 (13.3)	167 (44.5)
My skin itches	122 (32.6)	43 (11.5)	210 (56)
Water bothers my skin condition (bathing, washing hands)	270 (72)	26 (6.9)	79 (21.1)
My skin is irritated	197 (52.5)	63 (16.8)	115 (30.7)
My skin is sensitive	176 (47)	59 (15.7)	140 (37.3)
My skin condition bleeds	262 (69.9)	28 (7.5)	85 (22.7)
Functioning			
My skin condition affects how well I sleep	229 (61.1)	36 (9.6)	110 (29.3)
My skin condition makes it hard to work or do hobbies	198 (52.8)	32 (8.5)	145 (38.7)
My skin condition affects my social life	203 (54.2)	27 (7.2)	145 (38.7)
I tend to stay at home because of my skin condition	246 (65.6)	18 (4.8)	111 (29.6)
My skin condition affects how close I can be with those I love	244 (65.1)	26 (6.9)	105 (28)
I tend to do things by myself because of my skin condition	252 (67.2)	31 (8.3)	92 (24.6)
My skin condition makes showing affection difficult	266 (70.9)	28 (7.5)	81 (21.6)
My skin condition affects my interactions with others	227 (60.5)	30 (8.0)	118 (31.5)
My skin condition is a problem for the people I love	223 (59.5)	18 (4.8)	134 (35.8)
My skin condition affects my desire to be with people	236 (62.9)	31 (8.3)	108 (28.8)
My skin condition interferes with my sex life*	260 (79.3)	16 (4.9)	52 (15.8)
My skin condition makes me tired	241 (64.2)	26 (6.9)	108 (28.8)

*Frequencies and percent were calculated out of 328 participants as 47 subjects were not sexually active.

Furthermore, in the domain of symptoms, more than half of the respondents (56%) experienced skin itching all the time. Besides, about 44.5% of them reported that their skin condition frequently burns or stings. On the other hand, 72% of the participants reported that water rarely bothered their skin condition during bathing and hand washing. The mean (SD) score of the symptoms domain was 54.08 (20.5) out of 100. One-hundred eighty-nine of all the participants had severely impaired symptom domain.

In the functioning domain, about 38.7% of the respondents reported that their skin condition made it hard to work or do hobbies and it also affected their social life. Out of 328 participants who started a sexual life, 79.3% reported that their skin condition rarely interfered with their sex life. Moreover, 70.9% of the respondents reported that their skin condition made showing affection difficult. The mean (SD) score of the functioning domain was 46.89 (21.2) out of 100. A total of 216 participants had a severely impaired functionality (Fig. 1).

The overall mean (SD) score of the quality of life was 51.44 (18.9) out of 100. A total of 217 (57.9%) of the study participants had severely impaired QoL (Fig. 1).

Of the commonly seen skin diseases, patients with eczema, lichen planus, seborrhoeic dermatitis, psoriasis, acne vulgaris, and vitiligo had a severely impaired HRQoL outcome (Table 4). However, patients with pityriasis versicolor had no severely impaired HRQoL outcome.

**Table 4:** Mean scores of domains and total scale of Skindex-29 across the common skin diseases seen among the study participants (N = 375).

Skin disease	Emotion mean (SD)	Symptoms mean (SD)	Functioning mean (SD)	Total score mean (SD)
Eczema	**63.3 (19.8)**	**64.3 (18.2)**	**54.7 (21.9)**	**59.8 (17.6)**
Lichen planus	**57.0 (21.7)**	**52.9 (21.0)**	**43.3 (19.9)**	**50.2 (19.0)**
Seborrhoeic dermatitis	**55.1 (19.0)**	**52.7 (19.2)**	**40.6 (15.7)**	**48.5 (16.0)**
Psoriasis	**50.6 (15.9)**	**60.6 (15.0)**	**43.4 (18.4)**	**49.8 (14.5)**
Acne vulgaris	**61.4 (25.7)**	**59.0 (24.7)**	**50.5 (24.9)**	**55.9 (23.9)**
Pityriasis versicolor	**41.7 (20.8)**	32.7 (14.0)	30.8 (12.6)	34.9 (15.1)
Vitiligo	**60.0 (23.4)**	48.6 (21.0)	**49.4 (25.5)**	**52.7 (22.2)**

Values in bold were larger than the cut-off scores for severely impaired Health-related quality of life.

*SD* standard deviation.

### Determinants of health-related quality of life

Binary logistic regression analysis revealed that patients aged 41–59 years old (Crude Odds Ratio (COR) 1.93, 95% CI 1.16, 3.21), those with no formal education (COR 2.74, 95% CI 1.26, 5.98), patients with a primary educational level (COR 3.29, 95% CI 1.53, 7.06), and rural residents (COR 2.00, 95% CI 1.22, 3.29) were found to have a significant association with severely impaired QoL within the symptom domain (Table 5). Furthermore, those with a primary educational level (COR 3.19, 95% CI 1.20, 8.46) and chronic illness (COR 2.49, 95% CI 1.18, 5.25) were significant associates of severely impaired QoL within the emotion domain.

**Table 5:** Determinants, at bivariate level, of severely impaired health-related quality of life across the categories of socio-demographic and other background characteristics of outpatients attending dermatology clinic of Halibet National Referral Hospital, Eritrea, 2022.

Variables	Category	Symptoms COR (95%CI)	Emotions COR (95%CI)	Functioning COR (95%CI)	Total scale COR (95%CI)
Age	18–40	Ref.	Ref.	Ref.	Ref.
	41–59	1.93 (1.16, 3.21)*	1.20 (0.65, 2.20)	1.28 (0.77, 2.16)	1.37 (0.82, 2.30)
	60 and above	1.36 (0.70, 2.63)	0.60 (0.30, 1.22)	0.41 (0.21, 0.80)*	0.71 (0.37, 1.38)
Sex	Male	1.08 (0.72, 1.61)	1.32 (0.82, 2.12)	1.31 (0.87, 1.98)	1.23 (0.82, 1.85)
	Female	Ref.	Ref.	Ref.	Ref.
Educational level	No formal Education	2.74 (1.26, 5.98)*	1.29 (0.56, 2.98)	1.50 (0.70, 3.23)	1.95 (0.89, 4.26)
	Primary	3.29 (1.53, 7.06)**	3.19 (1.20, 8.46)*	1.65 (0.78, 3.46)	2.10 (0.99, 4.47)
	Junior	2.38 (0.91, 6.21)	2.21 (0.68, 7.17)	1.75 (0.66, 4.62)	1.84 (0.70, 4.85)
	Secondary	1.54 (0.91, 6.21)	1.63 (0.92, 2.90)	1.42 (0.84, 2.39)	1.42 (0.84, 2.40)
	Higher	Ref.	Ref.	Ref.	Ref.
Residence	Urban	Ref.	Ref.	Ref.	Ref.
	Rural	2.00 (1.22, 3.29)**	1.37 (0.76, 2.48)	1.68 (1.01, 2.79)*	1.90 (1.13, 3.18)*
Occupation	Employed	Ref.	Ref.	Ref.	Ref.
	Unemployed	1.14 (0.72, 1.83)	0.84 (0.49, 1.43)	0.81 (0.50, 1.30)	0.93 (0.58, 1.48)
	House wife	1.41 (0.79, 2.52)	1.00 (0.51, 1.97)	1.14 (0.63, 2.05)	1.39 (0.77, 2.53)
Presence of chronic illness	Yes	1.61 (0.95, 2.73)	2.49 (1.18, 5.25)*	2.09 (1.19, 3.69)*	2.07 (1.17, 3.64)*
	No	Ref.	Ref.	Ref.	Ref.

*CI* confidence interval; *COR* crude odds ratio; *Ref.* reference.

*p value < 0.05; **p value < 0.01;

In the functioning domain, patients aged 60 and above (COR 0.41, 95% CI 0.21, 0.80), rural residents (COR 1.68, 95% CI 1.01, 2.79), and those with chronic illness (COR 2.09, 95% CI 1.19, 3.69) were significantly associated with severely impaired QoL. Besides, being a rural resident (COR 1.90, 95% CI 1.13, 3.18) and presence of chronic illness (COR 2.07, 95% CI 1.17, 3.64) were significantly associated with severely impaired QoL within the overall HRQoL.

Multivariable logistic regression indicated that dermatology patients with a primary educational level were two times more likely to experience severely impaired symptom than those with a higher education (AOR 2.60, 95% CI 1.12, 6.03). Moreover, rural residents were approximately two times more likely to experience severely impaired symptom than urban residents (AOR 1.71, 95% CI 1.02, 2.86) (Table 6).

**Table 6:** Determinants, at multivariate level, of severely impaired health-related quality of life across the categories of socio-demographic and other background characteristics of outpatients attending dermatology clinic of Halibet National Referral Hospital, Eritrea, 2022.

Variables	Category	Symptoms AOR (95%CI)	Emotions AOR (95%CI)	Functioning AOR (95%CI)	Total scale AOR (95%CI)
Age	18–40	Ref.	Ref.	Ref.	Ref.
	41–59	1.34 (0.75, 2.40)	–	–	–
	60 and above	–	–	0.36 (0.18, 0.73)**	–
Sex	Male	–	–	–	–
	Female	Ref.	Ref.	Ref.	Ref.
Educational level	No formal education	2.41 (0.92, 6.29)	–	–	–
	Primary	2.60 (1.12, 6.03)*	2.94 (1.10, 7.86)*	–	–
	Junior	–	–	–	–
	Secondary	–	–	–	–
	Higher	Ref.	Ref.	Ref.	Ref.
Residence	Urban	Ref.	Ref.	Ref.	Ref.
	Rural	1.71 (1.02, 2.86)*	–	1.82 (1.08, 3.08)*	1.98 (1.18, 3.33)*
Occupation	Employed	Ref.	Ref.	Ref.	Ref.
	Unemployed	–	–	–	–
	House wife	–	–	–	–
Presence of chronic illness	Yes	–	2.36 (1.11, 4.99)*	2.21 (1.23, 3.98)**	2.16 (1.22, 3.82)**
	No	Ref.	Ref.	Ref.	Ref.

*AOR* adjusted odds ratio; *CI* confidence interval; *Ref.* reference.

*p value < 0.05; **p value < 0.01.

Dermatology patients with a primary educational level were approximately three times more likely to experience severely impaired emotions than those with a higher education (AOR 2.94, 95% CI 1.10, 7.86). Patients with chronic illnesses were two times more likely to experience severely impaired emotions than their counterparts (AOR 2.36, 95% CI 1.11, 4.99).

The elderly population was 64% less likely to experience severely impaired functioning than adults (AOR 0.36, 95% CI 0.18, 0.73). Moreover, rural residents were approximately two times more likely to experience severely impaired functioning than urban residents (AOR 1.82, 95% CI 1.08, 3.08). Patients with chronic illnesses were two times more likely to experience severely impaired functioning than their counterparts (AOR 2.21, 95% CI 1.23, 3.98).

In terms of overall health-related quality of life, rural residents were approximately two times more likely to experience severely impaired quality of life than urban residents (AOR 1.98, 95% CI 1.18, 3.33). Patients with chronic illnesses were two times more likely to experience severely impaired quality of life than their counterparts (AOR 2.16, 95% CI 1.22, 3.82) (Table 6).

A Chi-square test for trend analysis indicated that there was no significant association between the number of skin diseases in an individual patient and the overall quality of life outcome (χ^2^ = 2.34, *p* = 0.126) (Table 7).

**Table 7:** Association between number of skin diseases and health-related quality of life.

Description	Category	Number of skin disease in individual patient			Linear-by-linear association	
		1	2	3	χ^2^-value	*p* value
Quality of life severity	Severe	144	55	18	2.34	0.126
	Not severe	113	39	6		
	Total	257	94	24		

### Comparison of the domains of quality of life

**Figure 2:**
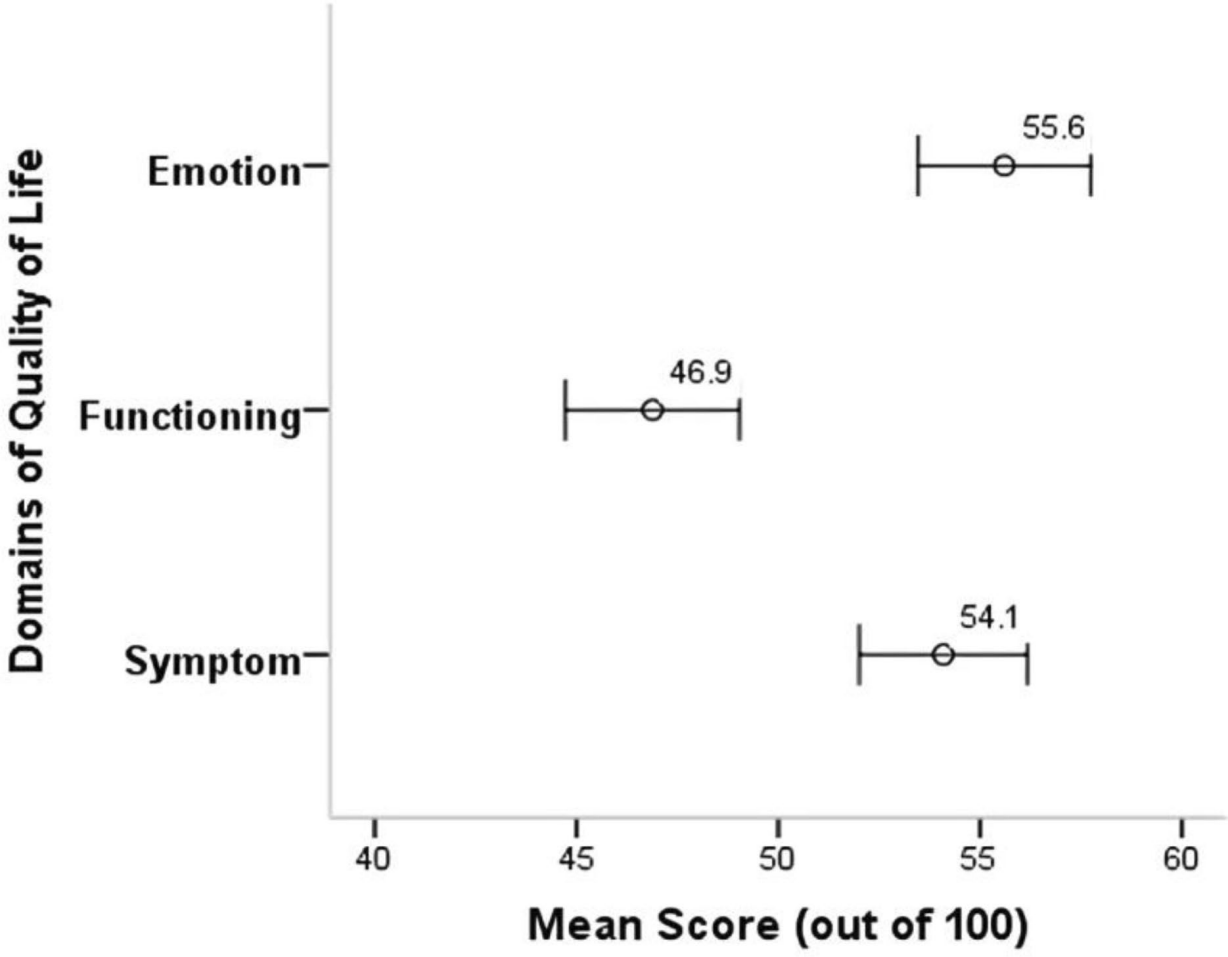
Comparison of the domains of quality of life.

Paired comparison of the domains of QoL revealed significantly higher mean score (*p* < 0.001) in emotion (M = 55.60, SD = 21.0) as compared to functioning (M = 46.89, SD = 21.2). On the other hand, significantly higher mean score (*p* < 0.001) was observed on symptom (M = 54.08, SD = 20.5) as compared to functioning (M = 46.89, SD = 21.2). However, the mean scores of emotion and symptom were not significantly different (MD = 1.51, 95% CI − 0.31, 3.33, *p* = 0.103) (Fig. 2).

## Discussion

Skin diseases compromise the quality of life of dermatology patients. The problems encountered from skin diseases include physical, psychological, and social consequences^21^. In this study, skin diseases severely impaired the health-related quality of life of study participants in terms of symptoms, emotions, and functioning. The majority of the dermatology outpatients experienced negative emotional effects from their skin diseases worrying about the severity, scarring, and worsening of their condition. Moreover, two out of five subjects felt depressed due to their skin condition. Skin diseases often lead to changes in appearance and negative body image which are associated with poor quality of life^16^. When patients face new symptoms or conditions, they react with fear and concern, impacting their well-being and treatment outcomes^17^. Dermatology patients are also more likely to experience psychiatric comorbidities compared to healthy individuals^22^. Our findings emphasize the importance of addressing adverse psychological outcomes associated with skin diseases which was in line with studies conducted in China^8^, South Korea^23^, and the UK^24^. Therefore, providing psychological support to dermatology patients is crucial due to the increased risk of psychiatric problems associated with skin diseases. This is supported by a UK study where 77% of the respondents would benefit from more psychological support^24^.

For about half of the respondents, skin diseases severely affected their quality of life in terms of symptoms. Most of them experienced constant itching, burning, or stinging. One out of five patients reported that water worsened their skin condition during bathing and hand washing. The symptoms associated with skin diseases can compromise the quality of life of patients thereby leading to subjective distress and emotional problems. In similar studies, physical symptom was found to be associated with greatest impact on their HRQoL^6,22^. In another study, patients with skin presentation of rashes compared to lesions were also found to have a severely impaired quality of life. Moreover, patients with extensive area of skin involvement suffer far greater than patients with skin cancer^25^.

More than half of the dermatologic outpatients experienced severely impaired functioning due to their skin condition. Several patients reported limitations in daily work and social life interaction, hobbies, and/or forced to stay at home. This aligns with findings of another study, that reported the skin conditions negatively affect patients’ routine activities and their social interactions and was associated with increased psychiatric morbidity^7^. Acquiring a primary educational level and being a rural resident were significantly associated with severely impaired quality of life within the symptom domain. Moreover, having a primary educational level and presence of systemic chronic illness were significantly associated with severely impaired emotional domain.

Being a rural resident and presence of systemic chronic illness were significantly associated with severely impaired functional domain and quality of life in general which is similar to findings reported from Belgium^26^. In other comparable study, individuals who are unemployed or living in rural areas were significantly affected^4^. Presence of other systemic chronic illness can aggravate the quality of life of patients which urges healthcare professionals and program managers to focus on a holistic approach that includes psychotherapy to improve the overall QoL of patients.

Moreover, dermatologic patients aged 18–40 years were significantly associated with severely impaired functioning likely due to unique challenges they face. The visibility and stigma of skin conditions can have a greater impact on socially and professionally active adults. Moreover, the psychosocial effects, such as poor body image and low self-confidence, could be more detrimental for adults with a well-established sense of identity and social dynamics. Hence, it is important to pay attention to optimal care that targets all aspects of health problems thereby improving their life conditions. As per the findings of the study, patients with atopic dermatitis (eczema), lichen planus, seborrhoeic dermatitis, psoriasis, acne vulgaris, and vitiligo were found to have significantly impaired overall QoL and had shown a profound psychosocial impairment. However, in a similar study conducted in Saudi Arabia, patients with eczematous dermatitis, connective tissue diseases and immunological disorders scored significantly higher in the symptom domain^27^.

The skin is certainly the most visible organ determining appearance and skin diseases have a detrimental effect on physical, emotional and functional domains of quality of life outcome and social interaction. The importance of this research and its findings are probably of specific interest to countries with socio-economic and cultural status similar to that of Eritrea. Program managers and policy makers are recommended to introduce psychosocial support activities in dermatologic clinics and organize continuous awareness-raising program on early treatment and understanding of skin disease. Besides, dermatologists and other healthcare professionals need to consider treating the soul along with the body to minimize impact of skin diseases.

### Limitation of the study

Due to the cross-sectional nature of the study, cause-effect relationship could not be established. This study was conducted in dermatology clinic of a tertiary care hospital, and thus, the findings could not be generalizable to other similar patients attending primary and secondary level health facilities.

## Conclusion

Skin diseases severely affected the emotional, functional and symptom domains of health-related quality of life of dermatologic patients. Overall, being a rural resident and having a chronic illness were significantly associated with severely impaired quality of life. To improve the quality of life of patients, program managers and health facility managers should consider introducing psychosocial support in dermatology clinics.

## Data Availability

The data used in this study and Tigrigna version of Skindex-29 are available from the corresponding author and can be accessed upon reasonable request.

## Abbreviations

AOR: Adjusted odds ratio
CI: Confidence interval
COR: Crude odds ratio
HRQoL: Health-related quality of life
IQR: Interquartile range
M: Mean
MD: Mean difference
Md: Median
MOH: Ministry of Health
SD: Standard deviation
SPSS: Statistical package for social sciences
QoL: Quality of life
WHO: World Health Organization
